# Crystal structure of (4*Z*)-1-(3,4-di­chloro­phen­yl)-4-[hy­droxy(4-methyl­phen­yl)methyl­idene]-3-methyl-4,5-di­hydro-1*H*-pyrazol-5-one

**DOI:** 10.1107/S160053681402114X

**Published:** 2014-09-30

**Authors:** Naresh Sharma, Sanjay Parihar, R. N. Jadeja, Rajni Kant, Vivek K. Gupta

**Affiliations:** a Post-Graduate Department of Physics & Electronics, University of Jammu, Jammu Tawi 180 006, India; b Department of Chemistry, Faculty of Science, The M.S. University of Baroda, Vadodara 390 002, India

**Keywords:** crystal structure, Schiff-base pyrazole derivative, hydrogen bonding, C—H⋯π inter­actions, aromatic π–π stacking

## Abstract

The title compound, C_18_H_14_Cl_2_N_2_O_2_, crystallizes with two mol­ecules, *A* and *B*, in the asymmetric unit. In mol­ecule *A*, the dihedral angles between the central pyrazole ring and pendant di­chloro­benzene and *p*-tolyl rings are 2.18 (16) and 46.78 (16)°, respectively. In mol­ecule *B*, the equivalent angles are 27.45 (16) and 40.45 (18)°, respectively. Each mol­ecule features an intra­molecular O—H⋯O hydrogen bond, which closes an *S*(6) ring and mol­ecule *A* also features a C—H⋯O inter­action. In the crystal, weak C—H⋯π interactions and aromatic π–π stacking [shortest centroid–centroid separation = 3.707 (2) Å] generate a three-dimensional network.

## Related literature   

For background to Schiff-base pyrazole derivatives, see: Jadeja *et al.* (2012 ▶). For a related structure, see: Abdel-Aziz *et al.* (2012 ▶).
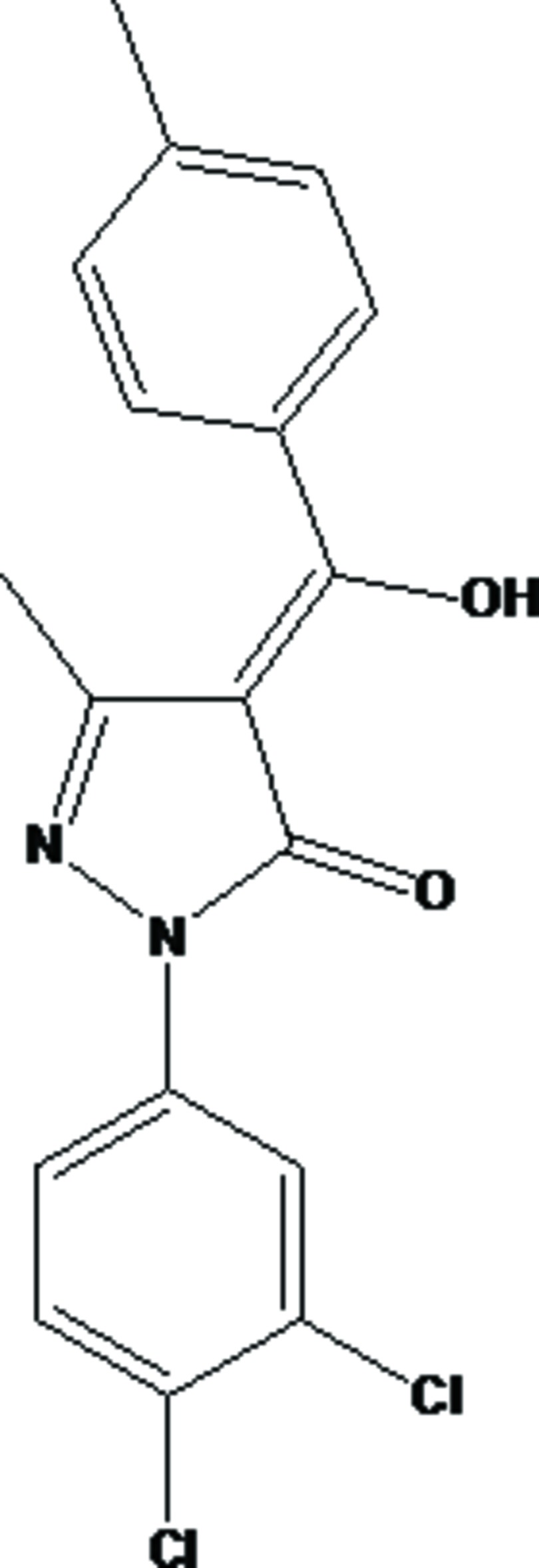


## Experimental   

### Crystal data  

C_18_H_14_Cl_2_N_2_O_2_
*M* *_r_* = 361.21
Triclinic, 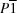

*a* = 7.5041 (5) Å
*b* = 15.4848 (9) Å
*c* = 15.5589 (10) Å
α = 71.963 (5)°
β = 80.731 (5)°
γ = 76.832 (5)°
*V* = 1665.87 (18) Å^3^
*Z* = 4
Mo *K*α radiation
μ = 0.40 mm^−1^
*T* = 293 K
0.30 × 0.20 × 0.20 mm

### Data collection  

Oxford Diffraction Xcalibur, Sapphire3 diffractometer
Absorption correction: multi-scan (Crys Alis RED; Agilent, 2013 ▶) *T*_min_ = 0.960, *T*_max_ = 1.000
12468 measured reflections
6536 independent reflections
3600 reflections with *I* > 2σ(*I*)
*R*_int_ = 0.037

### Refinement  

*R*[*F* ^2^ > 2σ(*F* ^2^)] = 0.054
*wR*(*F* ^2^) = 0.140
*S* = 0.99
6536 reflections
437 parameters
H-atom parameters constrained
Δρ_max_ = 0.24 e Å^−3^
Δρ_min_ = −0.24 e Å^−3^

### 

Data collection: *CrysAlis PRO* (Oxford Diffraction, 2010 ▶); cell refinement: *CrysAlis PRO*; data reduction: *CrysAlis PRO*; program(s) used to solve structure: *SHELXS97* (Sheldrick, 2008 ▶); program(s) used to refine structure: *SHELXL97* (Sheldrick, 2008 ▶); molecular graphics: *ORTEP-3 for Windows* (Farrugia, 2012 ▶); software used to prepare material for publication: *PLATON* (Spek, 2009 ▶).

## Supplementary Material

Crystal structure: contains datablock(s) I, New_Global_Publ_Block. DOI: 10.1107/S160053681402114X/hb7270sup1.cif

Structure factors: contains datablock(s) I. DOI: 10.1107/S160053681402114X/hb7270Isup2.hkl

Supporting information file. DOI: 10.1107/S160053681402114X/hb7270Isup3.cml

**Fig. 1.**
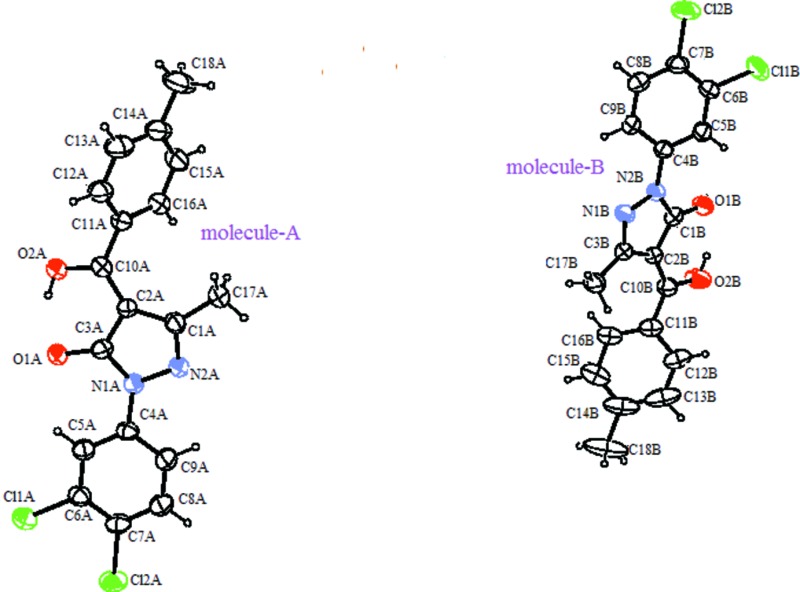
ORTEP view of the molecule with ellipsoids drawn at the 40% probability level. H atoms are shown as small spheres of arbitrary radii.

**Fig. 2.**
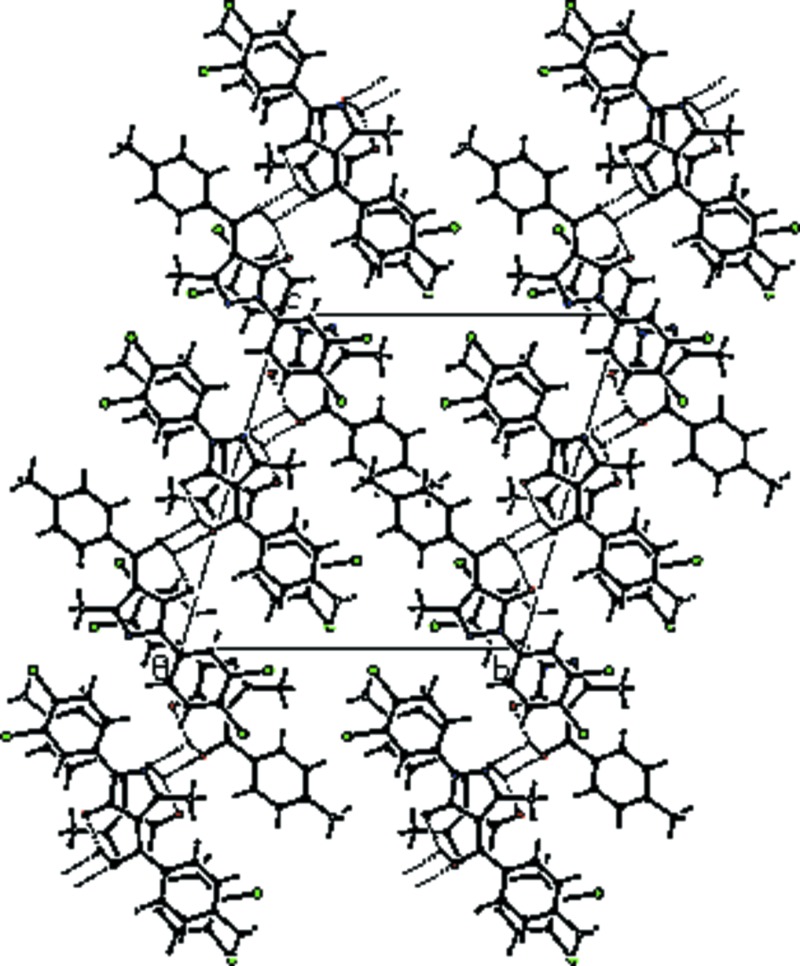
The packing arrangement of molecules viewed down the a axis.

ORTEP . DOI: 10.1107/S160053681402114X/hb7270fig1.tif
*ORTEP* view of the mol­ecule with ellipsoids drawn at the 40% probability level. H atoms are shown as small spheres of arbitrary radii.

a . DOI: 10.1107/S160053681402114X/hb7270fig2.tif
The packing arrangement of mol­ecules viewed down the *a* axis.

CCDC reference: 1025562

Additional supporting information:  crystallographic information; 3D view; checkCIF report

## Figures and Tables

**Table 1:** Hydrogen-bond geometry (Å, °) *Cg*5 is the centroid of the C11*A*/C12*A*–C16*A* ring.

*D*—H⋯*A*	*D*—H	H⋯*A*	*D*⋯*A*	*D*—H⋯*A*
O2*A*—H2*A*⋯O1*A*	0.82	1.78	2.549 (3)	155
C5*A*—H5*A*⋯O1*A*	0.93	2.27	2.904 (3)	125
O2*B*—H2*B*⋯O1*B*	0.82	1.76	2.527 (3)	154
C18*A*—H18*B*⋯*Cg*5^i^	0.96	2.85	3.616 (4)	136

Symmetry code: (i) 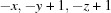.

## supplementary crystallographic information

### S1. Comment

4-Acyl pyrazolones can form a variety of Schiff bases and are reported to be superior reagents in biological, clinical and analytical applications (Jadeja *et al.*, (2012). In this article we are reporting synthesis and crystal structure of a new 4-acylpyrazolone derivative. The overall molecular geometry of the title compound is in good agreement with the corresponding values obtained in case of related structures (Abdel-Aziz *et al.*,2012). In the title compound C_18_H_14_Cl_2_N_2_O_2_, the dihedral angle between central pyrazole ring and dichlorophenyl ring is 2.23 (11) ° [molecule-A], between pyrazole ring [molecule-A] and *p*-tolyl ring [molecule-B] is 1.72 (11) ° and between dichlorophenyl ring [molecule-A] and *p*-tolyl ring [molecule-B] is 3.56 (11) °. The dihedral angles between pyrazole ring and dichlorophenyl ring [molecule-A], between pyrazole ring [molecule-A] and *p*-tolyl ring [molecule-B], between dichlorophenyl ring [molecule-A] and *p*-tolyl ring [molecule-B], shows that these rings are nearly co-planar to each other. The length of the double bond C3=O1 [1.291 (4) Å (molecule-A) and 1.279 (4) Å (molecule-B)] is significantly longer than that observed for carbonyl bonds, probably because atoms O1 and H2 are involved in strong intra-molecular O—H···O hydrogen bonds. The bond lengths of C6—Cl1 and C7—Cl2 [1.733 (3) Å and 1.733 (3) Å] for molecule-A, and [1.728 (4) Å and 1.730 (4) Å] for molecule-B, are quite comparable and agreed with the accepted value of 1.739 Å. The crystal structure features interactions of the type C—H···O, C—H···N, O—H···O and C—H···π. π - π interactions are also observed between the pyrazole and dichlorophenyl rings in the molecule-A at (1 - *x*, 2 - *y*, -*z*) [centroid–centroid seperation = 3.767 (2) Å], betwwen pyrazole rings in the molecule-B at (*x*, *y*, *z*) and (1 - *x*, -*y*, 1 - *z*) [centroid–centroid seperation = 3.797 (2) Å], between dichlorophenyl ring and *p*-tolyl ring (molecule-B) at (-*x*, -*y*, 1 - *z*) [centroid–centroid seperation = 3.707 (2) Å].

### S2. Experimental

1-(3,4-dichlorophenyl)-3-methyl-5-pyrazolone (24.2 g, 0.1 mol) and 80 ml of dry 1,4-dioxane were placed in a three necked 250 ml round bottom flask equipped with a stirrer, an addition funnel and a reflux condenser. The reaction mass was heated at 70 °C for 10 min. To the resulting yellow solution was added in small portions calcium hydroxide (14.82 g, 0.2 mol) and then toluoyl chloride (15.5 g, 0.1 mol) was added dropwise. During this addition, the whole mass was converted into a thick paste. After the complete addition, the reaction mixture was heated to reflux for 2 h. The yellowish mixture was cooled to room temperature and poured into a 250 ml solution of ice-cold hydrochloric acid (2 M) under stirring. The yellow precipitate was filtered, washed with water and dried in a vacuum. After drying a pale-yellow solid was obtained and recrystallized from an acetone-water mixture. (Yield 21.6 g m, 60%). Yellow blocks were obtained by the slow evaporation of the compound in acetone-water mixture (3–4 days).

(4*Z*)-1-(3,4,-dichlorophenyl)-4-(hydroxy(*p*-tolyl)methylene)-3-methyl-1*H*-pyrazol-5(4*H*)-one. ^1^H NMR (400 MHz, CDCl_3_, TMS): δ 2.15 (s, 3H), 2.48 (s, 3H), 7.34–7.36 (d, J = 8 Hz, 2H), 7.52–7.54 (d, J = 8.8 Hz, 1H), 7.57–7.59 (d, J = 8 Hz, 2H), 7.86–7.89 (dd, J = 2.4 Hz, 1H), 8.13–8.14 (d, J = 2.4 Hz, 1H). ^13^C NMR (CDCl_3_): δ 189.23, 163.56, 148.38, 143.19, 136.83, 133.24, 133.05, 130.66, 129.60, 129.18, 128.40, 121.54, 118.92, 103.86, 21.73, 16.20. ESI-MS: m/*z* 360.28 (calcd: m/*z* 360.04).

### S3. Refinement

All the H atoms were geometrically fixed and allowed to ride on their parent Carbon atoms, with C—H distances of 0.93–0.96 Å; and with *U*_iso_(H) = 1.2*U*_eq_(C), except for the methyl groups where *U*_iso_(H) = 1.5*U*_eq_(C),.

### Crystal data

**Table:**

C_18_H_14_Cl_2_N_2_O_2_	*Z* = 4
*M**_r_* = 361.21	*F*(000) = 744
Triclinic, *P*1	*D*_x_ = 1.440 Mg m^−^^3^
Hall symbol: -P 1	Mo *K*α radiation, λ = 0.71073 Å
*a* = 7.5041 (5) Å	Cell parameters from 2794 reflections
*b* = 15.4848 (9) Å	θ = 3.7–29.1°
*c* = 15.5589 (10) Å	µ = 0.40 mm^−^^1^
α = 71.963 (5)°	*T* = 293 K
β = 80.731 (5)°	Block, yellow
γ = 76.832 (5)°	0.30 × 0.20 × 0.20 mm
*V* = 1665.87 (18) Å^3^	

### Data collection

**Table:**

Oxford Diffraction Xcalibur, Sapphire3 diffractometer	6536 independent reflections
Radiation source: fine-focus sealed tube	3600 reflections with *I* > 2σ(*I*)
Graphite monochromator	*R*_int_ = 0.037
Detector resolution: 16.1049 pixels mm^-1^	θ_max_ = 26.0°, θ_min_ = 3.5°
ω scans	*h* = −9→9
Absorption correction: multi-scan (Crys Alis RED; Agilent, 2013)	*k* = −19→18
*T*_min_ = 0.960, *T*_max_ = 1.000	*l* = −18→19
12468 measured reflections	

### Refinement

**Table:**

Refinement on *F*^2^	Primary atom site location: structure-invariant direct methods
Least-squares matrix: full	Secondary atom site location: difference Fourier map
*R*[*F*^2^ > 2σ(*F*^2^)] = 0.054	Hydrogen site location: inferred from neighbouring sites
*wR*(*F*^2^) = 0.140	H-atom parameters constrained
*S* = 0.99	*w* = 1/[σ^2^(*F*_o_^2^) + (0.0477*P*)^2^] where *P* = (*F*_o_^2^ + 2*F*_c_^2^)/3
6536 reflections	(Δ/σ)_max_ = 0.001
437 parameters	Δρ_max_ = 0.24 e Å^−^^3^
0 restraints	Δρ_min_ = −0.24 e Å^−^^3^

### Special details

**Table:**

Experimental. Empirical absorption correction using spherical harmonics, implemented in SCALE3 ABSPACK scaling algorithm.
Geometry. All e.s.d.'s (except the e.s.d. in the dihedral angle between two l.s. planes) are estimated using the full covariance matrix. The cell e.s.d.'s are taken into account individually in the estimation of e.s.d.'s in distances, angles and torsion angles; correlations between e.s.d.'s in cell parameters are only used when they are defined by crystal symmetry. An approximate (isotropic) treatment of cell e.s.d.'s is used for estimating e.s.d.'s involving l.s. planes.
Refinement. Refinement of *F*^2^ against ALL reflections. The weighted *R*-factor *wR* and goodness of fit *S* are based on *F*^2^, conventional *R*-factors *R* are based on *F*, with *F* set to zero for negative *F*^2^. The threshold expression of *F*^2^ > σ(*F*^2^) is used only for calculating *R*-factors(gt) *etc*. and is not relevant to the choice of reflections for refinement. *R*-factors based on *F*^2^ are statistically about twice as large as those based on *F*, and *R*- factors based on ALL data will be even larger.

### Fractional atomic coordinates and isotropic or equivalent isotropic displacement parameters (Å^2^)

**Table:**

	*x*	*y*	*z*	*U*_iso_*/*U*_eq_	
Cl1A	0.89083 (13)	−0.27477 (5)	1.06691 (6)	0.0731 (3)	
Cl2A	0.68767 (12)	−0.25570 (6)	1.25713 (6)	0.0726 (3)	
O1A	0.8979 (3)	0.02319 (13)	0.82650 (14)	0.0605 (6)	
O2A	0.9047 (3)	0.15508 (14)	0.68000 (15)	0.0741 (7)	
H2A	0.9209	0.1037	0.7168	0.089*	
N1A	0.7441 (3)	0.07818 (15)	0.94796 (15)	0.0449 (6)	
N2A	0.6588 (3)	0.16407 (16)	0.96221 (16)	0.0519 (7)	
C1A	0.6813 (4)	0.22663 (19)	0.8851 (2)	0.0467 (7)	
C2A	0.7840 (4)	0.18608 (18)	0.81593 (18)	0.0424 (7)	
C3A	0.8170 (4)	0.0901 (2)	0.86035 (19)	0.0459 (7)	
C4A	0.7337 (4)	−0.00290 (19)	1.02012 (18)	0.0427 (7)	
C5A	0.8104 (4)	−0.08961 (19)	1.0096 (2)	0.0495 (8)	
H5A	0.8714	−0.0957	0.9542	0.059*	
C6A	0.7955 (4)	−0.1670 (2)	1.0822 (2)	0.0488 (7)	
C7A	0.7061 (4)	−0.1591 (2)	1.1653 (2)	0.0504 (8)	
C8A	0.6305 (4)	−0.0722 (2)	1.1750 (2)	0.0592 (9)	
H8A	0.5712	−0.0661	1.2306	0.071*	
C9A	0.6420 (4)	0.0049 (2)	1.1036 (2)	0.0577 (9)	
H9A	0.5885	0.0630	1.1107	0.069*	
C10A	0.8334 (4)	0.21605 (19)	0.7220 (2)	0.0488 (8)	
C11A	0.8110 (4)	0.31213 (19)	0.6644 (2)	0.0467 (7)	
C12A	0.7578 (4)	0.3345 (2)	0.5781 (2)	0.0584 (8)	
H12A	0.7369	0.2883	0.5566	0.070*	
C13A	0.7353 (4)	0.4249 (2)	0.5231 (2)	0.0682 (10)	
H13A	0.6979	0.4385	0.4654	0.082*	
C14A	0.7670 (4)	0.4950 (2)	0.5517 (3)	0.0630 (10)	
C15A	0.8276 (4)	0.4717 (2)	0.6364 (3)	0.0655 (10)	
H15A	0.8550	0.5175	0.6563	0.079*	
C16A	0.8487 (4)	0.3822 (2)	0.6926 (2)	0.0554 (8)	
H16A	0.8885	0.3688	0.7497	0.067*	
C17A	0.5964 (4)	0.32540 (19)	0.8800 (2)	0.0639 (9)	
H17A	0.5084	0.3276	0.9318	0.096*	
H17B	0.5357	0.3534	0.8255	0.096*	
H17C	0.6906	0.3584	0.8795	0.096*	
C18A	0.7404 (5)	0.5937 (2)	0.4933 (3)	0.0929 (14)	
H18A	0.7145	0.5964	0.4340	0.139*	
H18B	0.8504	0.6175	0.4883	0.139*	
H18C	0.6395	0.6303	0.5204	0.139*	
Cl1B	0.58908 (15)	1.43659 (6)	1.26432 (7)	0.0935 (4)	
Cl2B	0.70112 (15)	1.41790 (7)	1.06541 (6)	0.0910 (4)	
O1B	0.7689 (3)	1.13708 (13)	1.50039 (13)	0.0584 (6)	
O2B	0.7819 (3)	1.00850 (15)	1.64772 (15)	0.0779 (7)	
H2B	0.7686	1.0600	1.6109	0.094*	
N1B	0.7256 (3)	1.08466 (15)	1.38073 (16)	0.0459 (6)	
N2B	0.7082 (3)	1.00174 (16)	1.36756 (16)	0.0515 (6)	
C1B	0.7252 (4)	0.93871 (19)	1.4462 (2)	0.0452 (7)	
C2B	0.7553 (4)	0.97807 (19)	1.51388 (19)	0.0438 (7)	
C3B	0.7515 (4)	1.0732 (2)	1.4681 (2)	0.0474 (7)	
C4B	0.7189 (4)	1.16495 (19)	1.30626 (19)	0.0449 (7)	
C5B	0.6616 (4)	1.2518 (2)	1.3190 (2)	0.0516 (8)	
H5	0.6264	1.2581	1.3772	0.062*	
C6B	0.6568 (4)	1.3294 (2)	1.2447 (2)	0.0536 (8)	
C7B	0.7088 (4)	1.3211 (2)	1.1586 (2)	0.0555 (8)	
C8B	0.7640 (4)	1.2346 (2)	1.1462 (2)	0.0617 (9)	
H10	0.7956	1.2287	1.0877	0.074*	
C9B	0.7734 (4)	1.1561 (2)	1.2196 (2)	0.0542 (8)	
H9	0.8160	1.0978	1.2108	0.065*	
C10B	0.7744 (4)	0.9470 (2)	1.6079 (2)	0.0510 (8)	
C11B	0.7885 (4)	0.8533 (2)	1.6683 (2)	0.0541 (8)	
C12B	0.7131 (5)	0.8402 (3)	1.7580 (2)	0.0748 (11)	
H12	0.6513	0.8908	1.7780	0.090*	
C13B	0.7296 (6)	0.7528 (4)	1.8172 (3)	0.0990 (15)	
H13	0.6764	0.7452	1.8767	0.119*	
C14B	0.8236 (6)	0.6752 (3)	1.7909 (3)	0.0913 (14)	
C15B	0.9010 (5)	0.6895 (2)	1.7034 (3)	0.0800 (11)	
H14	0.9673	0.6389	1.6845	0.096*	
C16B	0.8847 (4)	0.7768 (2)	1.6414 (2)	0.0616 (9)	
H16	0.9382	0.7839	1.5820	0.074*	
C17B	0.7036 (4)	0.8437 (2)	1.4534 (2)	0.0627 (9)	
H17D	0.6329	0.8459	1.4063	0.094*	
H17E	0.6412	0.8180	1.5116	0.094*	
H17F	0.8226	0.8057	1.4470	0.094*	
C18B	0.8393 (7)	0.5792 (3)	1.8572 (3)	0.144 (2)	
H18D	0.7376	0.5526	1.8542	0.215*	
H18E	0.8379	0.5832	1.9176	0.215*	
H18F	0.9524	0.5411	1.8416	0.215*	

### Atomic displacement parameters (Å^2^)

**Table:**

	*U*^11^	*U*^22^	*U*^33^	*U*^12^	*U*^13^	*U*^23^
Cl1A	0.0999 (7)	0.0443 (5)	0.0620 (6)	−0.0064 (5)	−0.0022 (5)	−0.0046 (4)
Cl2A	0.0981 (6)	0.0631 (6)	0.0445 (5)	−0.0181 (5)	−0.0130 (4)	0.0070 (4)
O1A	0.0903 (15)	0.0338 (11)	0.0459 (13)	0.0011 (11)	0.0034 (11)	−0.0090 (10)
O2A	0.1251 (19)	0.0370 (12)	0.0475 (14)	−0.0068 (13)	0.0107 (13)	−0.0094 (11)
N1A	0.0555 (15)	0.0400 (14)	0.0373 (14)	−0.0056 (12)	−0.0058 (11)	−0.0102 (11)
N2A	0.0716 (17)	0.0387 (14)	0.0406 (15)	−0.0061 (13)	−0.0007 (13)	−0.0102 (12)
C1A	0.0531 (18)	0.0405 (17)	0.0485 (19)	−0.0086 (15)	−0.0073 (15)	−0.0145 (15)
C2A	0.0536 (17)	0.0358 (16)	0.0349 (16)	−0.0070 (14)	−0.0029 (13)	−0.0078 (13)
C3A	0.0533 (18)	0.0461 (18)	0.0360 (17)	−0.0118 (15)	−0.0029 (14)	−0.0076 (14)
C4A	0.0488 (17)	0.0422 (17)	0.0365 (17)	−0.0114 (14)	−0.0111 (13)	−0.0050 (13)
C5A	0.0574 (18)	0.0464 (18)	0.0414 (18)	−0.0074 (16)	−0.0077 (14)	−0.0079 (14)
C6A	0.0577 (18)	0.0435 (18)	0.0432 (18)	−0.0092 (15)	−0.0134 (14)	−0.0053 (14)
C7A	0.0570 (18)	0.0519 (19)	0.0370 (17)	−0.0121 (16)	−0.0153 (14)	0.0016 (15)
C8A	0.076 (2)	0.061 (2)	0.0369 (18)	−0.0109 (19)	−0.0061 (16)	−0.0097 (16)
C9A	0.076 (2)	0.054 (2)	0.0407 (19)	−0.0084 (17)	−0.0046 (16)	−0.0133 (16)
C10A	0.0589 (19)	0.0361 (16)	0.051 (2)	−0.0102 (15)	−0.0017 (15)	−0.0122 (15)
C11A	0.0514 (17)	0.0382 (17)	0.0452 (19)	−0.0081 (15)	−0.0002 (14)	−0.0069 (14)
C12A	0.077 (2)	0.052 (2)	0.045 (2)	−0.0161 (18)	−0.0095 (16)	−0.0071 (16)
C13A	0.072 (2)	0.068 (2)	0.052 (2)	−0.009 (2)	−0.0135 (17)	0.0013 (19)
C14A	0.058 (2)	0.049 (2)	0.064 (2)	−0.0074 (18)	0.0035 (18)	0.0030 (18)
C15A	0.076 (2)	0.043 (2)	0.077 (3)	−0.0179 (18)	0.002 (2)	−0.0169 (19)
C16A	0.069 (2)	0.0438 (19)	0.052 (2)	−0.0117 (17)	−0.0023 (16)	−0.0122 (16)
C17A	0.083 (2)	0.0429 (19)	0.059 (2)	−0.0045 (17)	0.0046 (18)	−0.0156 (16)
C18A	0.086 (3)	0.049 (2)	0.106 (3)	−0.004 (2)	0.001 (2)	0.019 (2)
Cl1B	0.1408 (9)	0.0441 (5)	0.0768 (7)	0.0147 (6)	−0.0136 (6)	−0.0120 (5)
Cl2B	0.1345 (8)	0.0651 (6)	0.0552 (6)	−0.0192 (6)	−0.0157 (6)	0.0122 (5)
O1B	0.0902 (15)	0.0445 (12)	0.0431 (13)	−0.0151 (11)	−0.0100 (11)	−0.0129 (10)
O2B	0.138 (2)	0.0606 (15)	0.0433 (14)	−0.0348 (15)	−0.0194 (14)	−0.0088 (12)
N1B	0.0605 (15)	0.0384 (14)	0.0374 (14)	−0.0066 (12)	−0.0060 (12)	−0.0101 (11)
N2B	0.0731 (17)	0.0385 (14)	0.0419 (15)	−0.0113 (13)	−0.0053 (13)	−0.0096 (12)
C1B	0.0510 (17)	0.0402 (17)	0.0408 (17)	−0.0068 (15)	0.0016 (14)	−0.0107 (14)
C2B	0.0504 (17)	0.0415 (17)	0.0355 (16)	−0.0078 (15)	0.0003 (13)	−0.0083 (13)
C3B	0.0527 (18)	0.0504 (19)	0.0377 (17)	−0.0095 (15)	0.0017 (14)	−0.0138 (15)
C4B	0.0461 (17)	0.0417 (17)	0.0402 (17)	−0.0022 (14)	−0.0052 (13)	−0.0060 (14)
C5B	0.0570 (19)	0.0499 (19)	0.0430 (18)	0.0006 (16)	−0.0048 (15)	−0.0137 (15)
C6B	0.0615 (19)	0.0392 (17)	0.050 (2)	0.0006 (15)	−0.0088 (15)	−0.0037 (15)
C7B	0.0605 (19)	0.051 (2)	0.0445 (19)	−0.0074 (17)	−0.0087 (15)	0.0007 (15)
C8B	0.078 (2)	0.063 (2)	0.0393 (19)	−0.0122 (19)	−0.0016 (16)	−0.0108 (17)
C9B	0.067 (2)	0.0478 (19)	0.0428 (19)	−0.0044 (16)	0.0006 (15)	−0.0137 (15)
C10B	0.0570 (18)	0.0500 (19)	0.0467 (19)	−0.0141 (16)	−0.0069 (15)	−0.0109 (15)
C11B	0.0544 (19)	0.057 (2)	0.047 (2)	−0.0188 (17)	−0.0099 (15)	−0.0006 (16)
C12B	0.080 (2)	0.093 (3)	0.044 (2)	−0.030 (2)	−0.0035 (18)	0.000 (2)
C13B	0.101 (3)	0.137 (4)	0.048 (2)	−0.061 (3)	−0.018 (2)	0.023 (3)
C14B	0.091 (3)	0.086 (3)	0.084 (3)	−0.048 (3)	−0.045 (3)	0.036 (3)
C15B	0.083 (3)	0.057 (2)	0.097 (3)	−0.024 (2)	−0.034 (2)	0.003 (2)
C16B	0.066 (2)	0.056 (2)	0.058 (2)	−0.0232 (18)	−0.0149 (17)	0.0033 (17)
C17B	0.091 (2)	0.0499 (19)	0.053 (2)	−0.0216 (18)	−0.0073 (18)	−0.0156 (16)
C18B	0.155 (4)	0.109 (4)	0.138 (5)	−0.076 (3)	−0.073 (4)	0.072 (3)

### Geometric parameters (Å, º)

**Table:**

Cl1A—C6A	1.733 (3)	Cl1B—C6B	1.728 (3)
Cl2A—C7A	1.733 (3)	Cl2B—C7B	1.730 (3)
O1A—C3A	1.291 (3)	O1B—C3B	1.279 (3)
O2A—C10A	1.282 (3)	O2B—C10B	1.302 (3)
O2A—H2A	0.8200	O2B—H2B	0.8200
N1A—C3A	1.357 (3)	N1B—C3B	1.356 (4)
N1A—N2A	1.406 (3)	N1B—N2B	1.398 (3)
N1A—C4A	1.409 (3)	N1B—C4B	1.410 (3)
N2A—C1A	1.302 (3)	N2B—C1B	1.311 (3)
C1A—C2A	1.441 (4)	C1B—C2B	1.439 (4)
C1A—C17A	1.500 (4)	C1B—C17B	1.485 (4)
C2A—C10A	1.405 (4)	C2B—C10B	1.411 (4)
C2A—C3A	1.415 (4)	C2B—C3B	1.420 (4)
C4A—C5A	1.384 (4)	C4B—C5B	1.381 (4)
C4A—C9A	1.395 (4)	C4B—C9B	1.384 (4)
C5A—C6A	1.380 (4)	C5B—C6B	1.383 (4)
C5A—H5A	0.9300	C5B—H5	0.9300
C6A—C7A	1.385 (4)	C6B—C7B	1.371 (4)
C7A—C8A	1.380 (4)	C7B—C8B	1.373 (4)
C8A—C9A	1.364 (4)	C8B—C9B	1.384 (4)
C8A—H8A	0.9300	C8B—H10	0.9300
C9A—H9A	0.9300	C9B—H9	0.9300
C10A—C11A	1.468 (4)	C10B—C11B	1.455 (4)
C11A—C12A	1.380 (4)	C11B—C16B	1.386 (4)
C11A—C16A	1.388 (4)	C11B—C12B	1.390 (4)
C12A—C13A	1.385 (4)	C12B—C13B	1.373 (5)
C12A—H12A	0.9300	C12B—H12	0.9300
C13A—C14A	1.372 (5)	C13B—C14B	1.390 (5)
C13A—H13A	0.9300	C13B—H13	0.9300
C14A—C15A	1.378 (5)	C14B—C15B	1.363 (6)
C14A—C18A	1.504 (4)	C14B—C18B	1.516 (5)
C15A—C16A	1.379 (4)	C15B—C16B	1.389 (4)
C15A—H15A	0.9300	C15B—H14	0.9300
C16A—H16A	0.9300	C16B—H16	0.9300
C17A—H17A	0.9600	C17B—H17D	0.9600
C17A—H17B	0.9600	C17B—H17E	0.9600
C17A—H17C	0.9600	C17B—H17F	0.9600
C18A—H18A	0.9600	C18B—H18D	0.9600
C18A—H18B	0.9600	C18B—H18E	0.9600
C18A—H18C	0.9600	C18B—H18F	0.9600
			
C10A—O2A—H2A	109.5	C10B—O2B—H2B	109.5
C3A—N1A—N2A	110.2 (2)	C3B—N1B—N2B	111.4 (2)
C3A—N1A—C4A	131.0 (2)	C3B—N1B—C4B	129.2 (2)
N2A—N1A—C4A	118.8 (2)	N2B—N1B—C4B	119.4 (2)
C1A—N2A—N1A	106.7 (2)	C1B—N2B—N1B	106.6 (2)
N2A—C1A—C2A	111.6 (2)	N2B—C1B—C2B	110.8 (3)
N2A—C1A—C17A	118.0 (2)	N2B—C1B—C17B	118.4 (3)
C2A—C1A—C17A	130.4 (3)	C2B—C1B—C17B	130.8 (3)
C10A—C2A—C3A	118.7 (2)	C10B—C2B—C3B	118.4 (3)
C10A—C2A—C1A	137.0 (3)	C10B—C2B—C1B	136.5 (3)
C3A—C2A—C1A	103.8 (2)	C3B—C2B—C1B	105.0 (3)
O1A—C3A—N1A	124.1 (3)	O1B—C3B—N1B	125.4 (3)
O1A—C3A—C2A	128.2 (3)	O1B—C3B—C2B	128.3 (3)
N1A—C3A—C2A	107.6 (2)	N1B—C3B—C2B	106.2 (3)
C5A—C4A—C9A	119.5 (3)	C5B—C4B—C9B	119.9 (3)
C5A—C4A—N1A	121.5 (2)	C5B—C4B—N1B	120.9 (2)
C9A—C4A—N1A	118.9 (2)	C9B—C4B—N1B	119.2 (2)
C6A—C5A—C4A	119.3 (3)	C4B—C5B—C6B	119.6 (3)
C6A—C5A—H5A	120.3	C4B—C5B—H5	120.2
C4A—C5A—H5A	120.3	C6B—C5B—H5	120.2
C5A—C6A—C7A	121.1 (3)	C7B—C6B—C5B	120.8 (3)
C5A—C6A—Cl1A	118.4 (2)	C7B—C6B—Cl1B	121.2 (2)
C7A—C6A—Cl1A	120.5 (2)	C5B—C6B—Cl1B	117.9 (2)
C8A—C7A—C6A	119.0 (3)	C6B—C7B—C8B	119.4 (3)
C8A—C7A—Cl2A	119.5 (2)	C6B—C7B—Cl2B	121.0 (2)
C6A—C7A—Cl2A	121.5 (2)	C8B—C7B—Cl2B	119.7 (2)
C9A—C8A—C7A	120.7 (3)	C7B—C8B—C9B	120.8 (3)
C9A—C8A—H8A	119.7	C7B—C8B—H10	119.6
C7A—C8A—H8A	119.7	C9B—C8B—H10	119.6
C8A—C9A—C4A	120.4 (3)	C4B—C9B—C8B	119.4 (3)
C8A—C9A—H9A	119.8	C4B—C9B—H9	120.3
C4A—C9A—H9A	119.8	C8B—C9B—H9	120.3
O2A—C10A—C2A	118.5 (3)	O2B—C10B—C2B	117.5 (3)
O2A—C10A—C11A	114.8 (3)	O2B—C10B—C11B	114.0 (3)
C2A—C10A—C11A	126.6 (3)	C2B—C10B—C11B	128.5 (3)
C12A—C11A—C16A	117.9 (3)	C16B—C11B—C12B	118.5 (3)
C12A—C11A—C10A	120.1 (3)	C16B—C11B—C10B	122.4 (3)
C16A—C11A—C10A	121.9 (3)	C12B—C11B—C10B	119.0 (3)
C11A—C12A—C13A	120.8 (3)	C13B—C12B—C11B	120.2 (4)
C11A—C12A—H12A	119.6	C13B—C12B—H12	119.9
C13A—C12A—H12A	119.6	C11B—C12B—H12	119.9
C14A—C13A—C12A	121.5 (3)	C12B—C13B—C14B	122.0 (4)
C14A—C13A—H13A	119.3	C12B—C13B—H13	119.0
C12A—C13A—H13A	119.3	C14B—C13B—H13	119.0
C13A—C14A—C15A	117.5 (3)	C15B—C14B—C13B	117.1 (4)
C13A—C14A—C18A	122.1 (4)	C15B—C14B—C18B	121.8 (5)
C15A—C14A—C18A	120.4 (4)	C13B—C14B—C18B	121.1 (5)
C14A—C15A—C16A	121.8 (3)	C14B—C15B—C16B	122.4 (4)
C14A—C15A—H15A	119.1	C14B—C15B—H14	118.8
C16A—C15A—H15A	119.1	C16B—C15B—H14	118.8
C15A—C16A—C11A	120.4 (3)	C11B—C16B—C15B	119.8 (3)
C15A—C16A—H16A	119.8	C11B—C16B—H16	120.1
C11A—C16A—H16A	119.8	C15B—C16B—H16	120.1
C1A—C17A—H17A	109.5	C1B—C17B—H17D	109.5
C1A—C17A—H17B	109.5	C1B—C17B—H17E	109.5
H17A—C17A—H17B	109.5	H17D—C17B—H17E	109.5
C1A—C17A—H17C	109.5	C1B—C17B—H17F	109.5
H17A—C17A—H17C	109.5	H17D—C17B—H17F	109.5
H17B—C17A—H17C	109.5	H17E—C17B—H17F	109.5
C14A—C18A—H18A	109.5	C14B—C18B—H18D	109.5
C14A—C18A—H18B	109.5	C14B—C18B—H18E	109.5
H18A—C18A—H18B	109.5	H18D—C18B—H18E	109.5
C14A—C18A—H18C	109.5	C14B—C18B—H18F	109.5
H18A—C18A—H18C	109.5	H18D—C18B—H18F	109.5
H18B—C18A—H18C	109.5	H18E—C18B—H18F	109.5
			
C3A—N1A—N2A—C1A	−0.6 (3)	C3B—N1B—N2B—C1B	−0.6 (3)
C4A—N1A—N2A—C1A	−177.3 (2)	C4B—N1B—N2B—C1B	178.0 (2)
N1A—N2A—C1A—C2A	−0.7 (3)	N1B—N2B—C1B—C2B	−0.3 (3)
N1A—N2A—C1A—C17A	177.6 (2)	N1B—N2B—C1B—C17B	177.2 (2)
N2A—C1A—C2A—C10A	172.6 (4)	N2B—C1B—C2B—C10B	176.5 (3)
C17A—C1A—C2A—C10A	−5.5 (6)	C17B—C1B—C2B—C10B	−0.6 (6)
N2A—C1A—C2A—C3A	1.6 (3)	N2B—C1B—C2B—C3B	1.1 (3)
C17A—C1A—C2A—C3A	−176.4 (3)	C17B—C1B—C2B—C3B	−176.0 (3)
N2A—N1A—C3A—O1A	−177.3 (3)	N2B—N1B—C3B—O1B	−178.9 (3)
C4A—N1A—C3A—O1A	−1.1 (5)	C4B—N1B—C3B—O1B	2.6 (5)
N2A—N1A—C3A—C2A	1.6 (3)	N2B—N1B—C3B—C2B	1.3 (3)
C4A—N1A—C3A—C2A	177.8 (3)	C4B—N1B—C3B—C2B	−177.2 (2)
C10A—C2A—C3A—O1A	4.0 (5)	C10B—C2B—C3B—O1B	2.4 (5)
C1A—C2A—C3A—O1A	177.0 (3)	C1B—C2B—C3B—O1B	178.8 (3)
C10A—C2A—C3A—N1A	−174.9 (3)	C10B—C2B—C3B—N1B	−177.8 (2)
C1A—C2A—C3A—N1A	−1.9 (3)	C1B—C2B—C3B—N1B	−1.4 (3)
C3A—N1A—C4A—C5A	2.3 (5)	C3B—N1B—C4B—C5B	−28.0 (4)
N2A—N1A—C4A—C5A	178.2 (2)	N2B—N1B—C4B—C5B	153.6 (3)
C3A—N1A—C4A—C9A	−177.0 (3)	C3B—N1B—C4B—C9B	150.9 (3)
N2A—N1A—C4A—C9A	−1.1 (4)	N2B—N1B—C4B—C9B	−27.4 (4)
C9A—C4A—C5A—C6A	−0.3 (4)	C9B—C4B—C5B—C6B	0.9 (5)
N1A—C4A—C5A—C6A	−179.6 (3)	N1B—C4B—C5B—C6B	179.9 (3)
C4A—C5A—C6A—C7A	−0.4 (5)	C4B—C5B—C6B—C7B	−0.2 (5)
C4A—C5A—C6A—Cl1A	179.4 (2)	C4B—C5B—C6B—Cl1B	−178.6 (2)
C5A—C6A—C7A—C8A	0.2 (5)	C5B—C6B—C7B—C8B	0.8 (5)
Cl1A—C6A—C7A—C8A	−179.5 (2)	Cl1B—C6B—C7B—C8B	179.1 (3)
C5A—C6A—C7A—Cl2A	−179.6 (2)	C5B—C6B—C7B—Cl2B	179.4 (2)
Cl1A—C6A—C7A—Cl2A	0.6 (4)	Cl1B—C6B—C7B—Cl2B	−2.3 (4)
C6A—C7A—C8A—C9A	0.6 (5)	C6B—C7B—C8B—C9B	−2.1 (5)
Cl2A—C7A—C8A—C9A	−179.6 (3)	Cl2B—C7B—C8B—C9B	179.3 (2)
C7A—C8A—C9A—C4A	−1.2 (5)	C5B—C4B—C9B—C8B	−2.1 (5)
C5A—C4A—C9A—C8A	1.1 (5)	N1B—C4B—C9B—C8B	178.9 (3)
N1A—C4A—C9A—C8A	−179.6 (3)	C7B—C8B—C9B—C4B	2.8 (5)
C3A—C2A—C10A—O2A	0.7 (5)	C3B—C2B—C10B—O2B	2.0 (4)
C1A—C2A—C10A—O2A	−169.3 (3)	C1B—C2B—C10B—O2B	−172.9 (3)
C3A—C2A—C10A—C11A	−179.7 (3)	C3B—C2B—C10B—C11B	−177.6 (3)
C1A—C2A—C10A—C11A	10.3 (6)	C1B—C2B—C10B—C11B	7.4 (6)
O2A—C10A—C11A—C12A	38.5 (4)	O2B—C10B—C11B—C16B	−141.9 (3)
C2A—C10A—C11A—C12A	−141.0 (3)	C2B—C10B—C11B—C16B	37.8 (5)
O2A—C10A—C11A—C16A	−139.0 (3)	O2B—C10B—C11B—C12B	33.8 (4)
C2A—C10A—C11A—C16A	41.5 (5)	C2B—C10B—C11B—C12B	−146.6 (3)
C16A—C11A—C12A—C13A	−2.7 (4)	C16B—C11B—C12B—C13B	−2.0 (5)
C10A—C11A—C12A—C13A	179.7 (3)	C10B—C11B—C12B—C13B	−177.9 (3)
C11A—C12A—C13A—C14A	0.8 (5)	C11B—C12B—C13B—C14B	1.2 (6)
C12A—C13A—C14A—C15A	1.9 (5)	C12B—C13B—C14B—C15B	0.7 (6)
C12A—C13A—C14A—C18A	−179.1 (3)	C12B—C13B—C14B—C18B	−179.7 (4)
C13A—C14A—C15A—C16A	−2.7 (5)	C13B—C14B—C15B—C16B	−1.6 (6)
C18A—C14A—C15A—C16A	178.3 (3)	C18B—C14B—C15B—C16B	178.7 (3)
C14A—C15A—C16A—C11A	0.8 (5)	C12B—C11B—C16B—C15B	1.1 (5)
C12A—C11A—C16A—C15A	2.0 (4)	C10B—C11B—C16B—C15B	176.8 (3)
C10A—C11A—C16A—C15A	179.5 (3)	C14B—C15B—C16B—C11B	0.8 (5)

### Hydrogen-bond geometry (Å, º)

Cg5 is the centroid of the C11A/C12A–C16A ring.

**Table:**

*D*—H···*A*	*D*—H	H···*A*	*D*···*A*	*D*—H···*A*
O2*A*—H2*A*···O1*A*	0.82	1.78	2.549 (3)	155
C5*A*—H5*A*···O1*A*	0.93	2.27	2.904 (3)	125
O2*B*—H2*B*···O1*B*	0.82	1.76	2.527 (3)	154
C18*A*—H18*B*···*Cg*5^i^	0.96	2.85	3.616 (4)	136

Symmetry code: (i) −*x*, −*y*+1, −*z*+1.
