# Supplementary figures and images for: Crystal structure of (4Z)-1-(3,4-di­chloro­phen­yl)-4-[hy­droxy(4-methyl­phen­yl)methyl­idene]-3-methyl-4,5-di­hydro-1H-pyrazol-5-one
Source: Acta Crystallogr Sect E Struct Rep Online. 2014 Sep 30;70(Pt 10):o1136–7. doi: 10.1107/S160053681402114X (PMC4257223; doi:10.1107/S160053681402114X)

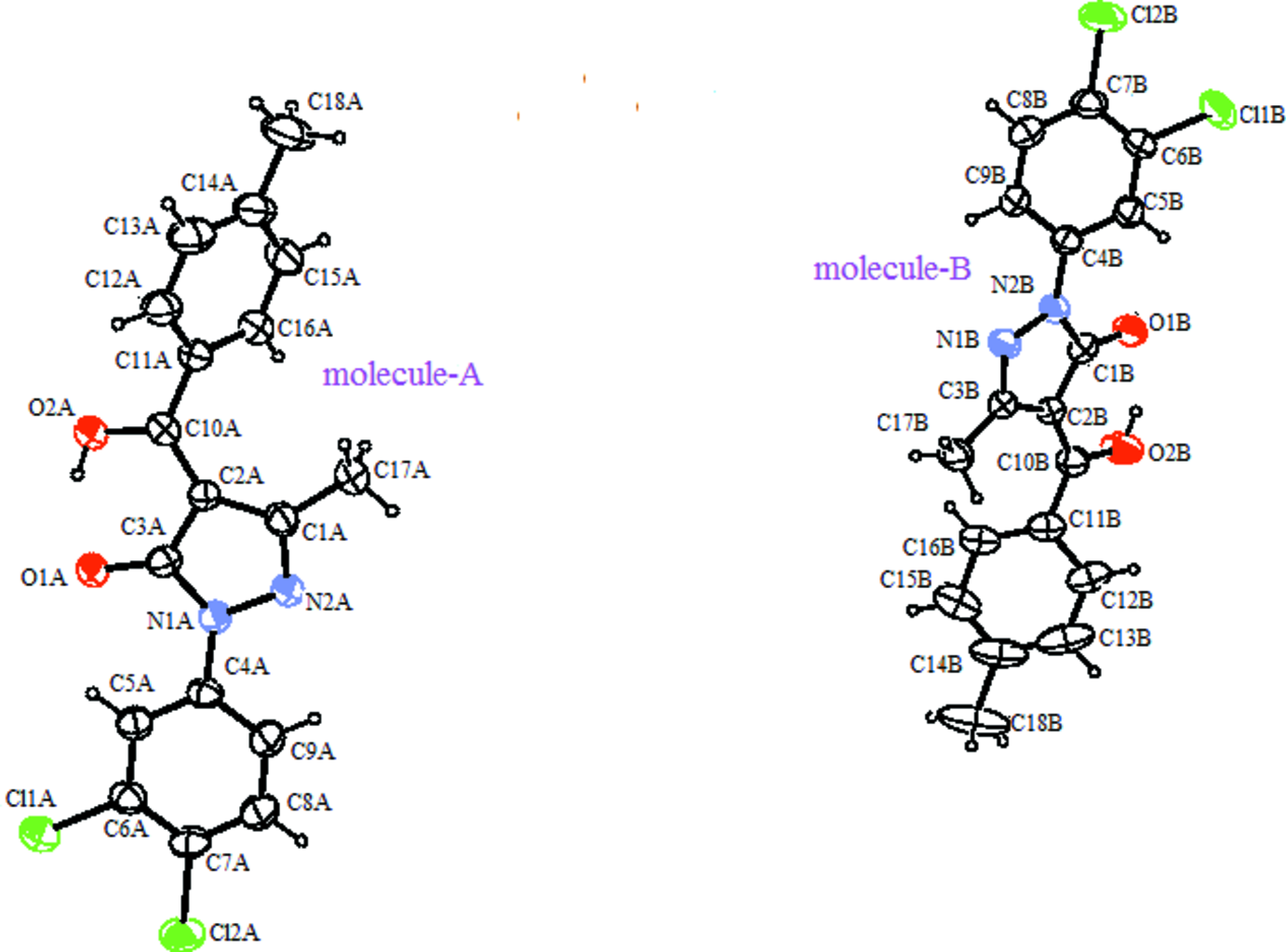

Supplement: Supplementary file 4 [file e-70-o1136-fig1.tif]

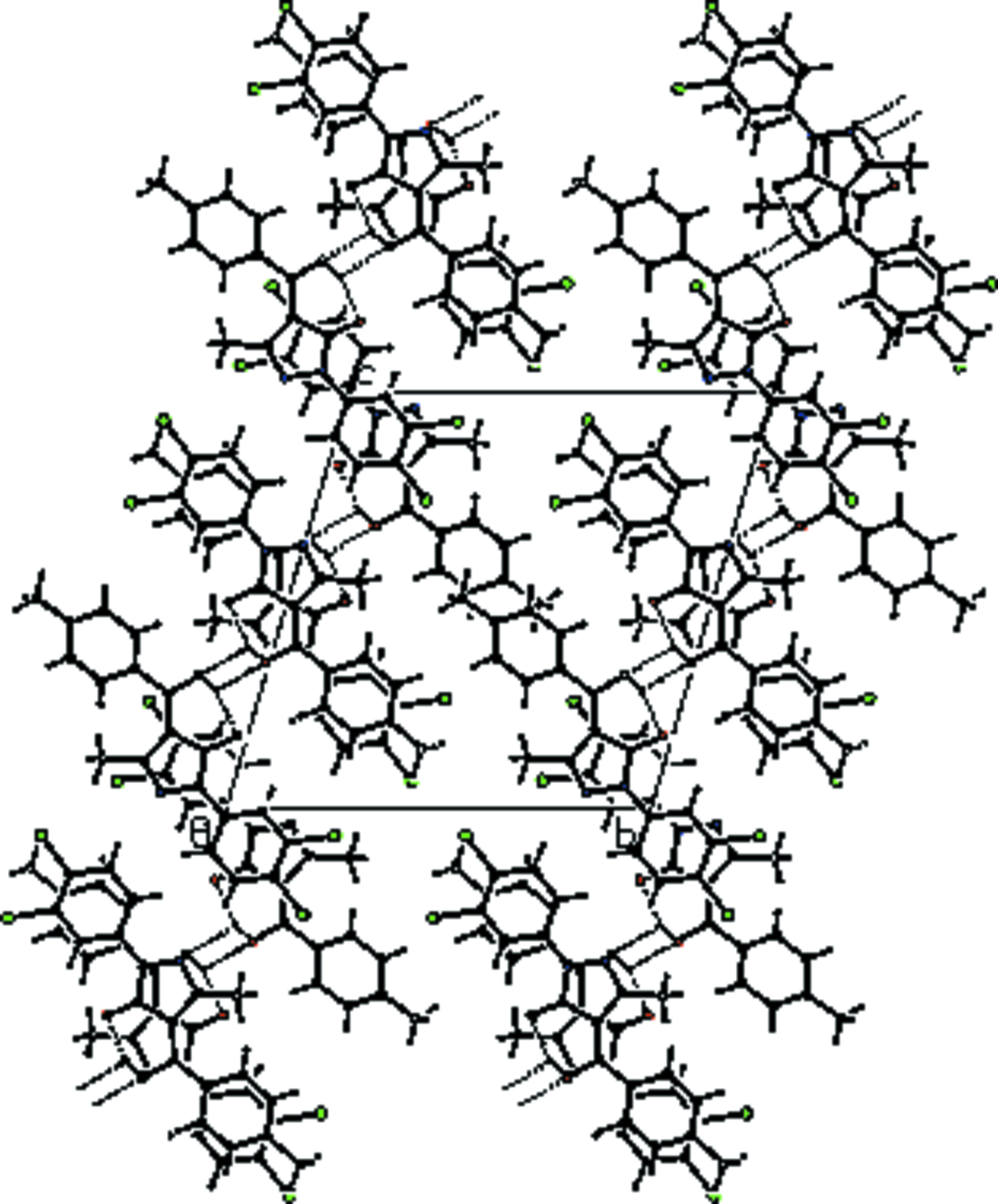

Supplement: Supplementary file 5 [file e-70-o1136-fig2.tif]
